# Trends and disparities in Non-Hodgkin Lymphoma related mortality in the United States, 1999–2020

**DOI:** 10.1371/journal.pone.0327809

**Published:** 2025-09-17

**Authors:** Muhammad Saad, Eliza Aisha, Muhammad Umer Sohail, Maryam Sajid, Syed Zaeem Ahmed, Zainab Siddiqua Ibrahim, Saad Ahmed Waqas, Haiqa Aamer, Fatima Aman Makda, Jazza Aamir, Muhammad Khalid Afridi, Raheel Ahmed

**Affiliations:** 1 Department of Medicine, Dow University of Health Sciences, Karachi, Pakistan; 2 National Heart and Lung Institute, Imperial College London, London, United Kingdom; The University of Alabama at Birmingham, UNITED STATES OF AMERICA

## Abstract

**Background:**

Non-Hodgkin Lymphoma (NHL) is the most prevalent hematological malignancy worldwide, making its mortality trends a critical public health concern. This study examines temporal trends in NHL-related mortality in the United States from 1999 to 2020, with a focus on sex-based, racial, and regional disparities.

**Methods:**

Data were obtained from the Centers for Disease Control and Prevention Wide-Ranging Online Data for Epidemiologic Research (CDC WONDER) database. Crude and age-adjusted mortality rates (AAMR) were analyzed and annual percentage changes (APCs) were calculated using Joinpoint regression to identify temporal trends.

**Results:**

Among 560,469 NHL-related deaths, AAMR declined from 12.86 (1999) to 8.26 per 100,000 (2020), with an increasing trend from 2018 to 2020 (APC: 0.89 [95% CI, −0.55 to 1.64]). Males consistently exhibited higher mortality rates compared to females (12.62 vs. 7.58), and NH White Americans exhibited the highest mortality among racial groups (10.34). Regional disparities were evident, with the Midwestern region recording the highest AAMR (10.64), and Minnesota, Nebraska, Iowa, Ohio and Vermont exhibiting the highest state-specific rates. Nonmetropolitan areas exhibited higher mortality rates than metropolitan areas (10.28 vs 9.62).

**Conclusion:**

While NHL-related mortality in the U.S. has declined overall, recent upward trends from 2018 to 2020, particularly among certain demographic and geographic subgroups, raise concerns. These findings highlight the need for further investigation into underlying factors and targeted interventions to address disparities and improve NHL outcomes.

## 1. Introduction

Non-Hodgkin lymphoma (NHL) is the seventh most common cancer and the sixth leading cause of cancer death in the United states (US) [1]. Forecasts for 2024 in the US alone predicted 80,620 new cases and 20,140 patient deaths, with half being men [2]. NHL exhibits high heterogeneity, which includes a wide range of origins, symptoms, treatment modalities, and results. The two most prevalent subtypes, diffuse large B-cell lymphoma (DLBCL) and follicular lymphoma, collectively comprise approximately 35% and 20% of all lymphomas, respectively [3,4].

NHL has been associated with immunosuppression which may stem from inherited conditions, infectious diseases, radiation therapy or organ transplantation [5–9] Individuals with congenital immunodeficiency syndromes face a significantly higher risk of NHL—28–49 times greater—than the general population, [5] while those with HIV have a risk approximately 100 times greater [10,11]

While NHL incidence rates nearly doubled between 1970 and 1990, advances in treatment have helped to stabilize the general population since the late 1990s [12] The prevalence of NHL underscores the need to better understand its impact on patient outcomes. However, trends in NHL-associated mortality remain inadequately explored. Recent work by Tan et al. (2024) examined NHL mortality in the US using Centers or Disease Control and Prevention’s Wide-ranging Online Data for Epidemiologic Research (CDC WONDER) data, focusing on underlying causes of death across sex, race, and geography [13]. Our study extends this by incorporating multiple cause of death data, which include both underlying and contributing causes, yielding a more accurate estimate of the true mortality burden. Reliance on underlying cause of death alone may underestimate cases where NHL contributed to death but was not the primary cause. We further stratify by place of death, providing new insight into care settings and palliative needs. Together, these additions enhance understanding of NHL mortality patterns and underscore the value of a dedicated analysis. This review aims to assess trends in NHL-associated mortality in the US from 1999 to 2020 and evaluate differences by sex, race, ethnicity, geographic region, and urbanization, using data from CDC WONDER. Our analysis aimed to investigate demographic and regional changes in NHL-related mortality among US adults aged 15 and older from 1999 to 2020 to identify at-risk individuals.

## 2. Methodology

### 2.1. Study setting and population

In this descriptive study, we examined death certificate data from the CDC WONDER database from 1999 to 2020 for NHL-related mortality among individuals aged 15 years or older, using codes from the International Statistical Classification of Diseases and Related Health Problems-10th Revision (ICD-10) as follows: C82, C83, C84, and C85 [14].

We focused on death certificates from the Multiple Cause of Death Public Use dataset, which allowed us to investigate cases where NHL played a role, either as a contributing factor or the primary cause of death. Institutional review board approval was not required, as we used a de-identified government-provided public-use dataset following Strengthening the Reporting of Observational Studies in Epidemiology (STROBE) guidelines [15]. For dataset preparation, analyses were restricted to U.S. resident deaths, and CDC WONDER default categorizations were applied; no additional recoding, filtering, or exclusions were performed. The mortality data are derived from death certificates filed in all 50 states and the District of Columbia. Deaths of nonresidents (including nonresident aliens, U.S. nationals living abroad, and residents of Puerto Rico, Guam, the Virgin Islands, and other U.S. territories) as well as fetal deaths are excluded. For race/ethnicity and place of death, small discrepancies between category-specific totals and overall deaths reflect records classified as ‘Unknown’ within CDC WONDER; these were retained as reported and no imputation was performed.

### 2.2. Data abstraction

The data abstracted for the study comprised population size, year, place of death, demographics, urban-rural classification, region, and states. The demographic data included sex, age, race/ethnicity, and location of death, which included medical facilities (outpatient, emergency room, inpatient, death on arrival, or status unknown), home, hospice, and nursing home/long-term care facility. The classification of race/ethnicity was based on non-Hispanic (NH) White, NH Black or African American, Hispanic or Latino, NH American Indian or Alaskan Native, and NH Asian or Pacific Islander.

We employed the Urban-Rural Classification Scheme from the National Center for Health Statistics to categorize the population into urban areas, which included large metropolitan regions (with populations exceeding 1 million) and medium/small metropolitan areas (with populations ranging from 50,000–999,999), as well as rural areas (with populations less than 50,000) based on the 2013 U.S. census classification [16]. In addition, we categorized regions into the Northeast, Midwest, South, and West, adhering to the definitions provided by the U.S. Census Bureau [9].

### 2.3. Statistical analysis

In this study, we assessed the NHL mortality rates from 1999 to 2020, considering various demographic factors such as gender, race, age, urbanization, and census data. Two types of mortality rates were calculated: crude mortality rates and age-adjusted mortality rates (AAMRs).

Crude mortality rates were computed by dividing the number of NHL-related deaths by the corresponding population of the US for each respective year. AAMRs, on the other hand, were standardized to the 2000 US population and accompanied by 95% confidence intervals.

To understand the national trends in NHL-related mortality over the years, we employed the Joinpoint Regression Program (Version 5.0.2, National Cancer Institute) [17]. This statistical tool allowed us to determine the annual percent change (APC) in AAMR, along with its associated 95% confidence interval. The APC method is instrumental in identifying significant changes in AAMR over time, employing log-linear regression models to detect temporal variations. To determine whether the APCs were increasing or decreasing, we evaluated their statistical significance by comparing them to the null hypothesis of zero change. We employed a two-tailed t-test with a significance threshold of P < 0.05 to establish statistical significance.

## 3. Results

A total of 560,469 NHL-related deaths occurred between 1999 and 2020 among adolescents and adults (≥15 years old) **(Supplementary Figure 1, Supplementary Table 1** in S1 File). Information on the location of death was available for 559,079 deaths. Of these, most deaths occurred within medical facilities (44.58%), followed by the decedent’s home (30.27%), followed by nursing home/long term care (14.41%), followed by hospice facilities (6.09%), and others (0.25%). Annual trends in NHL-related mortality stratified by place of death are shown in **Supplementary Table 2 in** S1 File.

### 3.1. Annual trends for NHL-related AAMR

**Fig 1:**
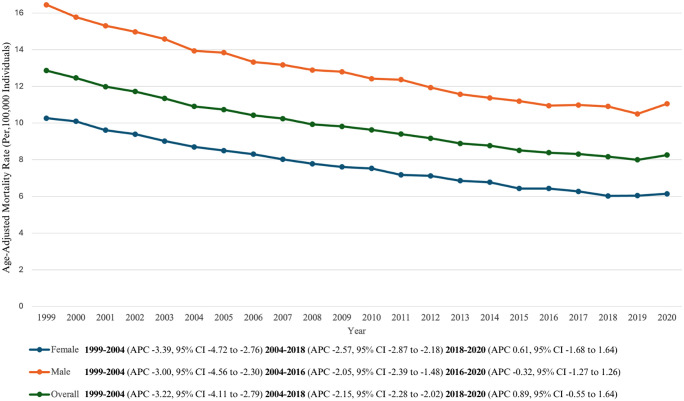
Non-Hodgkin Lymphoma related AAMRs per 100,000 stratified by sex in the United States from 1999 to 2020.

The AAMR for NHL-related deaths in adults was 12.86 in 1999 and 8.26 in 2020. (Table 1) The overall AAMR decreased steeply between 1999 and 2004 (APC: −3.22 [95% CI, −4.11 to −2.79]), followed by a relatively less steep decrease from 2004 till 2018 (APC: −2.15 [95% CI, −2.28 to −2.02]). From 2018 to 2020, an increase in AAMR was noted (APC: 0.89 [95% CI, −0.55 to 1.64]). Visual trends are illustrated in **Fig 1** with comprehensive data present in **Supplementary Table 3** and **Supplementary Table 4 in** S1 File.

**Table 1:** Frequency and age adjusted rates per 100,000 in adults with NHL stratified by sex, race, census region and location of death.

	Deaths	Population	AAMR 1999 (95% CI)	AAMR 2020 (95% CI)
**Overall**	560,469	5,409,649,211	12.86 (12.70-13.01)	8.26 (8.14-8.38)
**Sex**				
**Male**	308,815	2,633,991,877	16.45 (16.18-16.72)	11.06 (10.88-11.24)
**Female**	251,654	2,775,657,334	10.27 (10.09-10.45)	6.14 (6.03-6.26)
**Non-Hispanic Race**				
**NH American Indian/Alaska Native**	2,050	42,536,402	7.26 (5.58–9.29)	6.66 (5.51–7.80)
**NH Asian or Pacific Islander**	13,435	287,111,166	7.76 (6.97–8.56)	5.53 (5.17–5.89)
**NH Black or African American**	38,881	659,572,487	9.68 (9.24–10.12)	6.03 (5.76–6.31)
**NH White**	470,986	3,644,401,586	13.42 (13.25–13.59)	8.84 (8.72–8.96)
**Hispanic Race**	34,046	776,027,570	9.61 (9.03–10.20)	7.13 (6.82–7.43)
**Census Region**				
**Northeast**	110,588	990,000,000	13.20 (12.87-13.54)	8.22 (7.99-8.45)
**Midwest**	137,821	1,170,000,000	13.86 (13.54-14.19)	9.22 (8.99-9.45)
**South**	194,849	2,000,000,000	12.19 (11.94-12.44)	7.91 (7.75-8.07)
**West**	117,211	1,250,000,000	12.54 (12.21-12.87)	8.07 (7.86-8.28)
**Place of Death**				
**Medical facility**	235,477	–	–	–
**Hospice facility**	34,123	–	–	–
**Nursing home/long term care**	80,764	–	–	–
**Decedent’s home**	169,641	–	–	–

-Represents data unavailable from CDC WONDER.

Non-Hispanic (NH)

Non-Hodgkin Lymphoma (NHL)

Age-Adjusted Mortality Rate (AAMR)

### 3.2. NHL-Related AAMR Stratified by Sex

During the study period, male AAMR was consistently higher than female AAMR (overall AAMR men: 12.62 [95% CI, 12.57 to 12.66]; women: 7.58 [95% CI, 7.55 to 7.61]).

In 1999, the AAMR for men was 16.45 (95% CI: 16.12 to 16.72), which decreased to 13.94 in 2004 (APC: −3.00[95% CI, −4.56 to −2.30]), followed by a lesser decrease to 10.95 in 2016 (APC: −2.05 [95% CI, −2.39 to −1.48]) and 11.06 in 2020 (APC: −0.32[95% CI, −1.27 to 1.26]).

Similarly, the AAMR for women was 10.27 in 1999 (95% CI: 10.09 to 10.45), which decreased to 8.70 in 2004 (APC: −3.39[95% CI, −4.72 to −2.76]), followed by a continued decrease up to 2018 (APC: −2.57 [95% CI, −2.87 to −2.18]), and an increase from 2018 to 2020 (APC: 0.61[95% CI, −1.68 to 1.64]).

Visual trends are illustrated in Fig 1 with comprehensive data present in **Supplementary Table 3** and **Supplementary Table 4 in** S1 File.

### 3.3 NHL-Related AAMR Stratified by Race/Ethnicity

Throughout the study period, NH White Americans had the highest overall AAMR among all ethnicities (overall AAMR: 10.34 [95% CI, 10.32 to 10.37]). They were followed by Hispanics or Latinos (7.53 [95% CI, 7.44–7.61]), NH Black Americans (7.03 [95% CI, 6.95 to 7.10]), NH American Indian or Alaska Native (6.54 [95% CI, 6.25 to 6.84]) and NH Asian or Pacific Islander populations (6.04 [95% CI, 5.94 to 6.14]).

From 1999 to 2018, all ethnicities experienced a decline in AAMR values. The AAMR for NH American Indian or Alaska Native continued to decrease till 2020 (APC: −1.44 [95% CI, −2.32 to −0.46]) as well as the AAMR for NH Asian or Pacific Islander (APC: −1.72 [95% CI, −1.96 to −1.44]). The AAMR for NH Black Americans decreased steeply until 2005 (APC: −3.77 [95% CI, −7.81 to −2.30]), followed by a lesser decrease till 2020 (APC: −1.40 [95% CI, −1.77 to −0.21]). AAMR for NH White Americans decreased steeply till 2004 (APC:-3.06 [95% CI,-3.98 to −2.64]) followed by a lesser decrease till 2018 (APC:-2.12 [95% CI, −2.24 to −1.97]) with Hispanic or Latinos following a similar trend (APC: −1.77 [95% CI, −3.24 to −1.03]). The AAMR for NH Whites increased from 2018 to 2020 (APC: 1.33 [95% CI, −0.17 to 2.16]). A similar trend was noted for Hispanic or Latinos with a much steeper increase from 2018 to 2020 (APC: 3.11 [95% CI, −1.61 to 5.79]).

**Fig 2:**
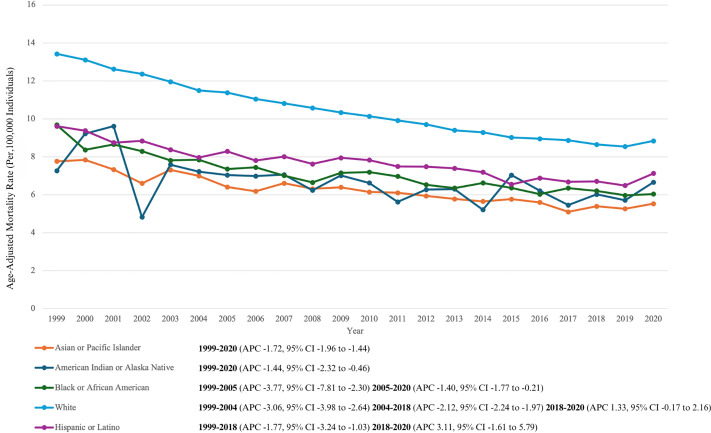
Non-Hodgkin Lymphoma related AAMRs per 100,000 stratified by race in the United States from 1999 to 2020.

Visual trends are illustrated in Fig 2 with comprehensive data present in **Supplementary Table 3** and **Supplementary Table 5 in** S1 File.

### 3.4. NHL-Related AAMR Stratified by Geographic Region

A noticeable variation in AAMR was observed among states, with values ranging from 8.17 (95% CI, 7.90–8.44) in Nevada to 11.22 (95% CI, 11.01–11.42) in Minnesota. States in the upper 90th percentile of NHL-related deaths included Minnesota, Nebraska, Iowa, Ohio and Vermont, (**Supplementary Table 7 in** S1 File) which had approximately 1.4 times the AAMRs compared to states in the lower 10th percentile of NHL-related deaths, comprising Georgia, Hawaii, District of Columbia, New Mexico and Nevada.

Over the course of the study period, the highest mortality was observed in the Midwestern region (AAMR: 10.64[95% CI, 10.59 to 10.70]), followed by the Northeastern (AAMR: 9.82 [95% CI, 9.76 to 9.88]), Western (AAMR: 9.49[95% CI, 9.43 to 9.54]), and Southern (AAMR: 9.27[95% CI, 9.23 to 9.31]) regions. Comprehensive data is available in **Supplementary Figure 2** and **Supplementary Table 8 in** S1 File.

A state-wise table highlighting regional disparities in NHL-related mortality is presented in **Supplementary Table 7** and **Supplementary Table 8 in** S1 File.

Metropolitan and Nonmetropolitan areas exhibited comparable overall AAMRs of 9.62 (95% CI: 9.60–9.65) and 10.28 (95% CI: 10.22–10.35), respectively. From 1999 to 2000, metropolitan areas showed higher NHL-related AAMRs compared to nonmetropolitan areas. This was followed by a reversal in trends, with nonmetropolitan areas having higher AAMRs than their metropolitan counterparts until 2020. Between 1999 and 2004, there was a steep reduction in the AAMR in metropolitan areas (APC: −3.30 [95% CI, −4.41 to −2.83]), followed by a more gradual decline until 2016 (APC: −2.29 [95% CI, −2.48 to −2.04]) and from 2016 to 2020 (APC: −0.78 [95% CI, −1.46 to 0.80]). For nonmetropolitan areas, a continuous decline in AAMR from 1999 to 2018 was seen (APC: −2.02 [95% CI, −2.27 to – 1.86]), followed by an increasing trend until 2020 (APC: 3.25 [95% CI, −0.76 to 4.95]).

Visual trends are illustrated in **Supplementary Figure 3 in** S1 File with comprehensive data available in **Supplementary Table 3** and **Supplementary Table 6 in** S1 File.

### 3.5. NHL as an Underlying Cause of Death

Out of 560,469 NHL-related deaths, 457,143 listed NHL as the underlying cause of death. In the analysis limited to cases where NHL was exclusively identified as the underlying cause of death, NHL mortality in the overall population was 4.98% in 1999, which then dipped to 4.87% in 2001. Subsequently, there was a steeper decrease to 4.57% in 2004, followed by a less steep decline to 4.41% in 2020. Males had higher NHL mortality throughout the study period when compared to females (AAMR male: 10.1 [95% CI, 10.06 to 10.14]; AAMR female: 6.29 [95% CI, 6.27 to 6.32]). **(Supplementary Table 9 in** S1 File)

## 4. Discussion

**Fig 3:**
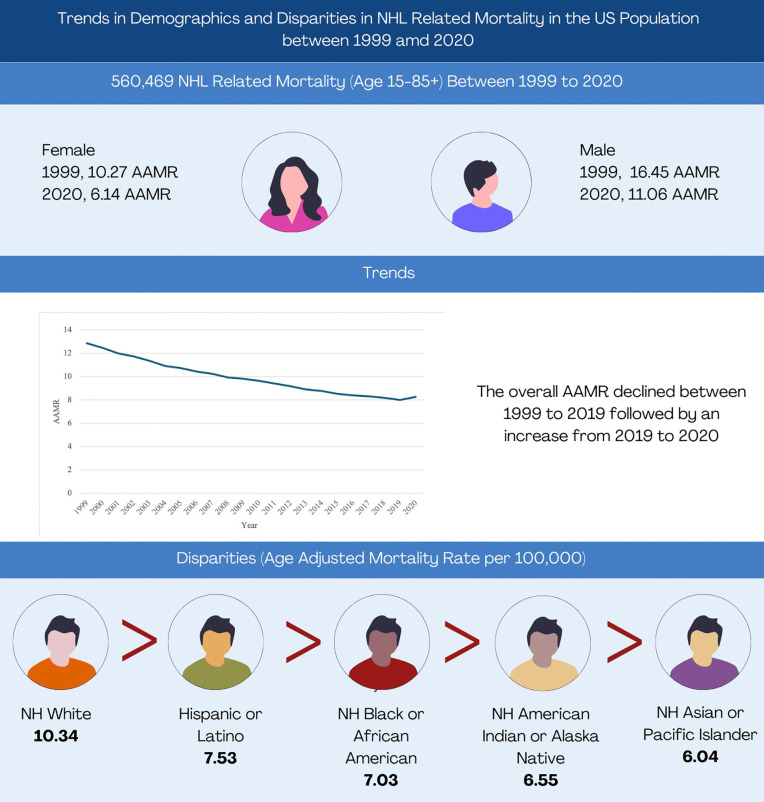
Central Illustration.

Our study, examining NHL-related mortality trends from 1999 to 2020, is the first to comprehensively analyze CDC WONDER data by gender, race/ethnicity, region, and place of death. In this 22-year analysis of mortality data, we report several key findings. First, we observed a sharp decline in mortality rates from 1999 to 2018, followed by an increase until 2020. Second, among all racial groups, NH White Americans had the highest NHL-related AAMR, which experienced an upward trajectory after 2018. Third, we observed variations in AAMR geographically, with states in the upper 90th percentile (Minnesota, Nebraska, Iowa, Ohio and Vermont) displaying nearly 1.4 times the AAMR compared to those in the lower 10th percentile (Georgia, Hawaii, District of Columbia, New Mexico and Nevada). Notably, from 1999 to 2000, metropolitan areas exhibited higher NHL-related AAMRs compared to nonmetropolitan areas, a trend that subsequently reversed, with nonmetropolitan areas surpassing their metropolitan counterparts until 2020. These findings hold substantive implications for public health policy formulation and underscore the necessity of addressing the nuanced temporal and geographical dynamics observed in NHL-related mortality trends. (Fig 3)

Our study offers a broader and more nuanced view of NHL-related mortality by using multiple cause of death data, rather than limiting the analysis to underlying cause of death as done in the study by Tan et al.[13] This distinction is important, as multiple cause of death also captures deaths where NHL contributed to mortality but was not the primary cause—particularly relevant during the COVID-19 pandemic. Notably, reliance on underlying cause of death data alone may underestimate the true mortality burden of chronic illnesses like NHL. This concern has been previously highlighted by Minhas et al., who demonstrated that underlying cause of death-based analyses can underrepresent disease impact by excluding cases where a condition is a contributing factor rather than the primary cause [18]. By incorporating multiple cause of death data and performing additional stratifications,such as by place of death, and sociodemographic subgroups,our study offers a more nuanced understanding of NHL-related mortality trends and disparities in the US. Lastly, unlike previous studies, ours includes stratification by both place of death and state, offering a deeper understanding of regional differences in mortality trends.

From 2018 to 2020, a modest increase in AAMR was noted, marking the first uptick in nearly two decades. The AAMR for NHL listed as the Multiple Cause of Death rose from 8.00 in 2019 to 8.26 in 2020. Notably, this increase coincides with the onset of the COVID-19 pandemic. During this period, the number of deaths with NHL as the underlying cause of death remained relatively stable. In fact, the AAMR for NHL as the underlying cause of death, slightly declined from 6.38 in 2019 to 6.21 in 2020. This pattern is consistent with findings from previous studies, suggesting that the overall rise in NHL-related mortality is largely attributable to COVID-19–related deaths in patients with NHL, where NHL was a contributing factor rather than the primary cause of death [13]. This trend appears to be linked to the heightened susceptibility of NHL patients to COVID-19 infection, resulting in deaths from COVID-19 where NHL is listed as a contributing factor [19,20].

Race-wise stratification of NHL-related mortality trends revealed an overall decrease in all races until 2018, followed by an increase in AAMR for the White and Hispanic or Latino populations from 2018 to 2020. Nevertheless, Black individuals and Hispanics have some of the lowest AAMRs, which may be attributable to their low incidence rates. Although AAMR remains a valuable summary measure, it should be interpreted cautiously, as it conflates disease incidence with case-fatality and thus cannot reveal whether observed mortality differences reflect fewer new diagnoses or differences in survival after diagnosis. Studies have highlighted that NH White Americans not only have the highest incidence of NHL but also experienced a 65.9% increase between 2014 and 2018, whereas the rise among Black adults was only 7.8% [21]. Once diagnosed, however, prognosis is poorest for Black patients, with five-year relative survival of 69.5% compared with 77.6% in White patients [21]. Advanced-stage presentation is more common in Black individuals, and socioeconomic barriers, including lower income and limited insurance, further delay treatment [22,23]. It is also likely that access to high-quality oncology centers and persistent under-representation in trials also widen survival gaps [24]. A review of phase I oncology trials found that White/Caucasians made up 84.2% of U.S. participants, while Black individuals, Asians, and Hispanics represented only 7.3%, 3.4%, and 2.8%, respectively [25].

Gender-wise stratification of NHL-related mortality trends revealed an overall decrease in both male and female populations. Nevertheless, AAMRs for men remained notably higher compared to women, consistent with global NHL trends [26–28]. While large prospective and epidemiologic studies consistently indicate male sex as an indicator for worse survival, the underlying reasons remain inadequately understood. The elevated risk among men may be attributed to a higher prevalence of specific risk factors, including HIV and chemical exposures [29]. Additionally, certain NHL subtypes, such as Burkitt lymphoma, diffuse large B cell lymphoma (DLBCL), Sezary syndrome, and various T cell lymphomas, exhibit a pronounced male/female incidence ratio [30,31].

Differences in mortality trends may also be elucidated by gender-associated responses to therapy. A study demonstrated that women exhibit a superior response to R-CHOP therapy [32]. It was also found that the addition of rituximab to therapy showed no response difference between young males and females. However, among older adults, women benefited more, attributed to the lower clearance rate of rituximab in older females than males [33]. A UK-based phase II study suggested a significant correlation between sex and response to lenalidomide in patients with relapsed/refractory mantle cell lymphoma, indicating that female patients are more sensitive to lenalidomide than their male counterparts [34].

Pregnancy emerges as a potentially protective factor in NHL. Studies have established an inverse relationship between pregnancy and the risk of B-cell NHL [35]. A decreased risk across different NHL subtypes with oral contraceptive use has also been observed, particularly among women who initiated use at an earlier age (<25 years) [36,37]. However, the molecular mechanisms underlying the influence of sex hormones on NHL development remain incompletely understood. Potential mechanisms include the impact of estrogen on the host immune response. 17β-estradiol decreases interleukin 6 (IL6) production by mononuclear cells, resulting in lower serum IL6 levels [38]. Elevated serum IL6 levels correlate with reduced complete response and diminished overall survival in DLBCL patients [39]. This suggests a protective effect of estrogen by downregulating IL6 levels. Another proposed mechanism involves the direct effect of estrogen on lymphocytes expressing estrogen receptors [40]. There is evidence for the anti-proliferative effect of estrogen on lymphoid cells through estrogen receptor β (ERβ) signaling, inhibiting the proliferation of T cell lymphoma and Burkitt lymphoma cells in vitro [41].

The reasons behind the higher mortality rates observed in non-metropolitan areas are multifaceted. Tao et al. has shown that a lack of insurance is associated with increased mortality from non-Hodgkin lymphoma. Given that non-metropolitan regions tend to have lower rates of insurance coverage compared to their metropolitan counterparts, this finding supports our observation of elevated mortality in non-metropolitan areas. Additionally, socioeconomic status has been identified as a significant factor influencing mortality rates [42]. It’s conceivable that non-metropolitan areas in the U.S. face lower socioeconomic status levels compared to metropolitan areas, a trend observed in other countries, which could contribute to the higher mortality rates in non-metropolitan regions [43]. Lastly, the prevalence of obesity, a known risk factor for NHL, tends to be higher in non-metropolitan areas, further exacerbating mortality rates in these regions [28,44].

Over the past two decades, there has been a decline in NHL incidence and mortality, attributed to advancements in treatment and diagnosis. Mortality from AIDS related NHL, which makes up 4.6% of all NHL cases, has decreased in recent era due to improvements in HAART regimens and increased utilization of HAART in the population [45]. Rituximab, which was introduced in 1998 and is used in combination with chemotherapy, has greatly improved response rates, length of remission, and survival in patients with follicular lymphoma and diffuse large B-cell lymphoma [46–53]. Key studies, such as the US intergroup trial, highlight the advantages of rituximab-based regimens. For instance, the 3-year failure-free survival rate was significantly higher at 53% for patients treated with R-CHOP compared to 46% for those treated with CHOP alone [53]. However, the exact mechanisms of rituximab’s action are still under investigation, and its limited efficacy in B-cell NHL results in poor outcomes in certain NHL subtypes [54,55].

### Limitations

There are various limitations to this analysis. Firstly, the actual prevalence of NHL as a cause of death may be distorted due to the dependence on ICD-10 codes and death certificates. The veracity of the reported data could be compromised by this reliance, which has the potential to either skew or exclude deaths associated to NHL. Secondly, information regarding the disease’s initial characteristics, the therapies administered, and other particulars of the ailment is lacking, therefore, it was not possible to analyze differences in treatment modalities, access, or related aspects of care. Third, the CDC WONDER database is subject to underreporting and reporting lag, particularly during the COVID-19 period, which may affect the accuracy of recent mortality trends. These constraints should be considered when interpreting the findings. Lastly, the analysis lacks a comprehensive perspective due to the absence of data regarding laboratory parameters, vital signs, and other diagnostic investigations.

### Conclusion

Between 1999 and 2018, the AAMR for NHL decreased consistently, indicating advances in treatment efficacy and diagnostic measures. However, AAMR increased substantially between 2018 and 2020, possibly owing to increased mortality caused by the COVID-19 infection during the pandemic. Nevertheless, disparities in mortality rates persist across demographic and geographical lines. Notably, males, NH Whites, individuals residing in the Midwestern region, and those living in nonmetropolitan areas of the US exhibit disproportionately higher AAMRs among those aged 15 and older. Addressing these discrepancies require strategies and interventions aimed at reducing disparities in mortality within various population subsets.

## Supporting information

S1 File S**upplementary Figure 1.** Flow Diagram representing the inclusion and exclusion criteria of the study. **Supplementary Figure 2.**NHL-related AAMRs per 100,000 stratified by census region in the United States from 1999 to 2020. **Supplementary Figure 3.**NHL-related AAMRs per 100,000 stratified by urbanization in the United States from 1999 to 2020. **Supplementary Table 1.** Overall and sex-stratified NHL-related deaths in the United States from 1999 to 2020. **Supplementary Table 2.** NHL-related mortality stratified by place of death in the United States from 1999 to 2020. **Supplementary Table 3.** Summary APCs of NHL-related AAMR per 100,000 in the United States from 1999 to 2020. **Supplementary Table 4.** Overall and sex-stratified NHL-related AAMR per 100,000 in the United States from 1999 to 2020. **Supplementary Table 5.** NHL-related AAMR per 100,000 stratified by Race in the United States from 1999 to 2020. **Supplementary Table 6.** NHL-related AAMR per 100,000 stratified by Urban-Rural classification in the United States from 1999 to 2020. **Supplementary Table 7.** NHL-related AAMR per 100,000 stratified by state in the United States from 1999 to 2020. **Supplementary Table 8.** NHL-related AAMR per 100,000 stratified by census region in the United States from 1999 to 2020.**Supplementary Table 9.** NHL-related deaths per 100,000 stratified by top 15 underlying causes of death in the United States from 1999 to 2020.
(DOCX)
