# Expression of Concern: Trends and disparities in Non-Hodgkin Lymphoma related mortality in the United States, 1999–2020

**DOI:** 10.1371/journal.pone.0346387

**Published:** 2026-04-07

**Authors:** The PLOS One Editors

The *PLOS One* Editors issue this Expression of Concern to alert readers that this article [1] was identified as one of a series of submissions for which we have concerns about authorship and adherence to the journal’s first and second publication criteria [2]. We regret that these issues were not addressed prior to the article’s publication.
