# Supplementary material for: Trends and disparities in Non-Hodgkin Lymphoma related mortality in the United States, 1999–2020
Source: PLoS One. 2025 Sep 17;20(9):e0327809. doi: 10.1371/journal.pone.0327809 (PMC12443298; doi:10.1371/journal.pone.0327809)
Supplement: S1 File — Supplementary Figure 1. Flow Diagram representing the inclusion and exclusion criteria of the study. Supplementary Figure 2.NHL-related AAMRs per 100,000 stratified by census region in the United States from 1999 to 2020. Supplementary Figure 3.NHL-related AAMRs per 100,000 stratified by urbanization in the United States from 1999 to 2020. Supplementary Table 1. Overall and sex-stratified NHL-related deaths in the United States from 1999 to 2020. Supplementary Table 2. NHL-related mortality stratified by place of death in the United States from 1999 to 2020. Supplementary Table 3. Summary APCs of NHL-related AAMR per 100,000 in the United States from 1999 to 2020. Supplementary Table 4. Overall and sex-stratified NHL-related AAMR per 100,000 in the United States from 1999 to 2020. Supplementary Table 5. NHL-related AAMR per 100,000 stratified by Race in the United States from 1999 to 2020. Supplementary Table 6. NHL-related AAMR per 100,000 stratified by Urban-Rural classification in the United States from 1999 to 2020. Supplementary Table 7. NHL-related AAMR per 100,000 stratified by state in the United States from 1999 to 2020. Supplementary Table 8. NHL-related AAMR per 100,000 stratified by census region in the United States from 1999 to 2020.Supplementary Table 9. NHL-related deaths per 100,000 stratified by top 15 underlying causes of death in the United States from 1999 to 2020. (DOCX) [file pone.0327809.s001.docx]

**Supplementary Figure 1**. Flow Diagram representing the inclusion and exclusion criteria of the study.

**
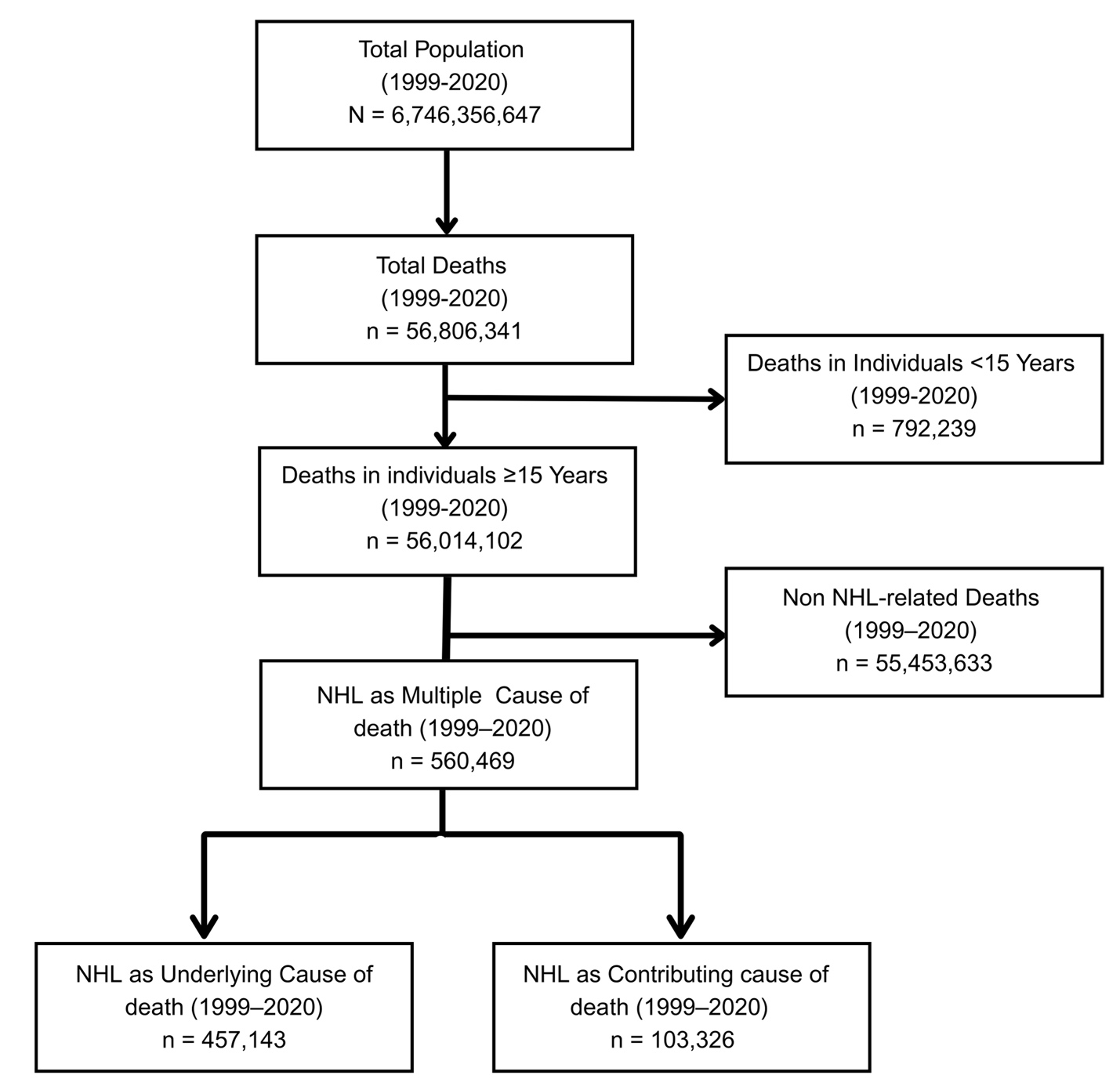
**

**Supplementary Figure 2**.NHL-related AAMRs per 100,000 stratified by census region in the United States from 1999 to 2020.


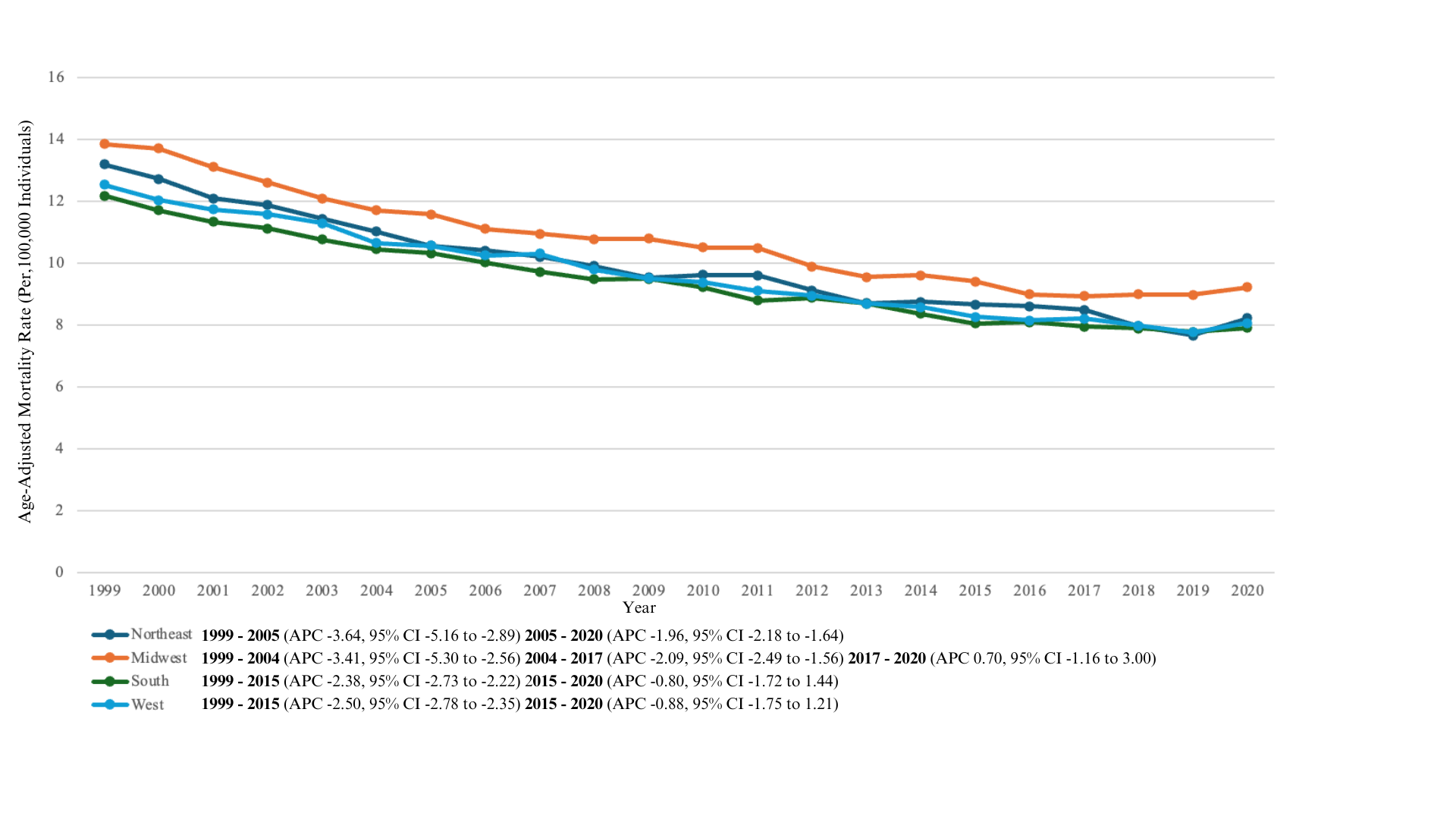


**Supplementary Figure 3**.NHL-related AAMRs per 100,000 stratified by urbanization in the United States from 1999 to 2020.


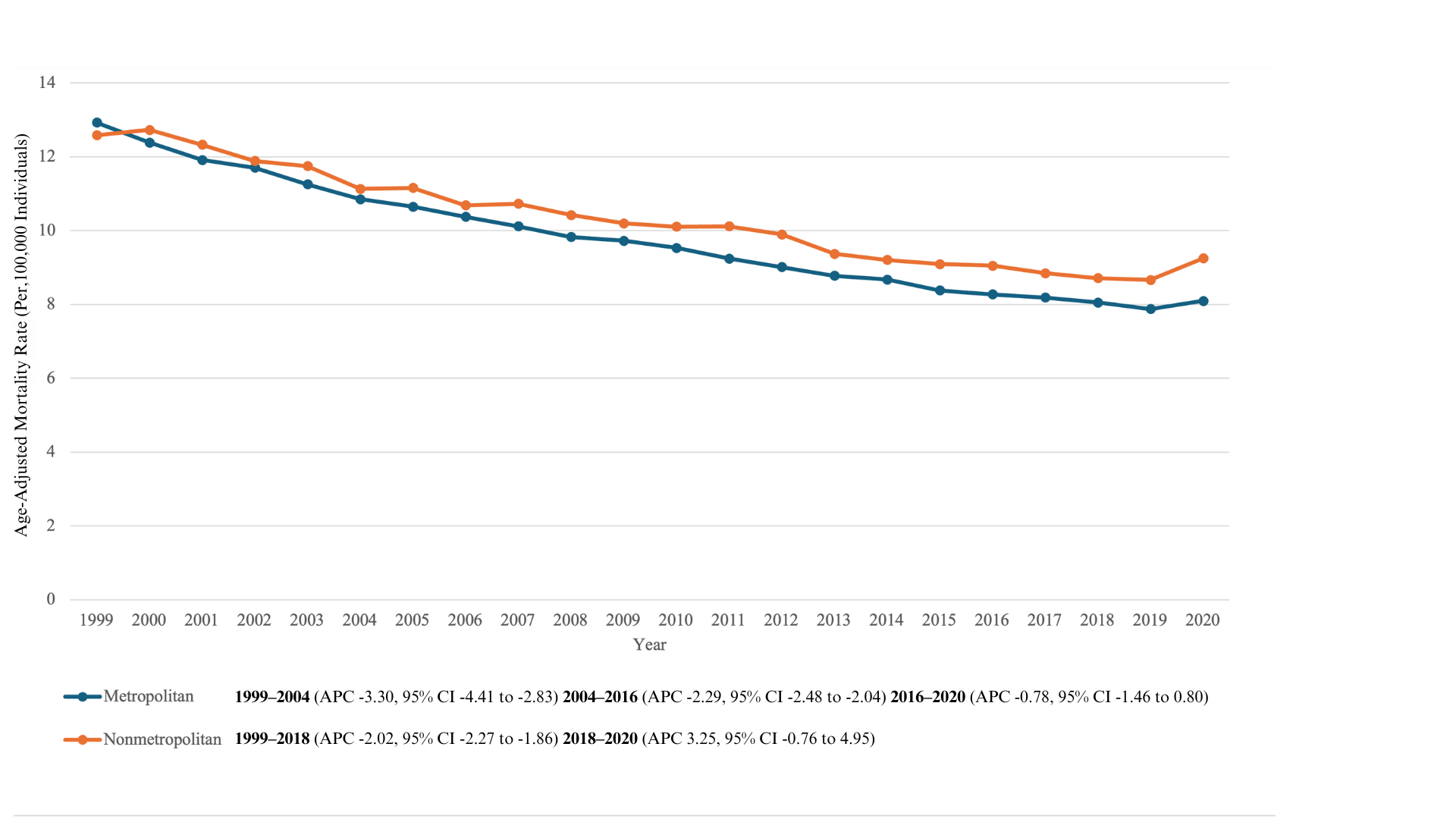


**Supplementary Table 1.** Overall and sex-stratified NHL-related deaths in the United States from 1999 to 2020.

| **Year** | **Overall** | **Male** | **Female** | **NH American Indian or Alaska Native** | **NH Asian or Pacific Islander** | **NH Black or African American** | **NH White** | **Hispanic or Latino** | **Population** |
| --- | --- | --- | --- | --- | --- | --- | --- | --- | --- |
| **1999** | 27,687 | 14,761 | 12,926 | 72 | 415 | 1,953 | 23,952 | 1,220 | 219,084,800 |
| **2000** | 27,087 | 14,215 | 12,872 | 91 | 439 | 1,678 | 23,625 | 1,187 | 221,168,531 |
| **2001** | 26,508 | 14,057 | 12,451 | 101 | 437 | 1,770 | 22,969 | 1,168 | 224,518,698 |
| **2002** | 26,330 | 13,995 | 12,335 | 55 | 433 | 1,726 | 22,807 | 1,235 | 227,062,163 |
| **2003** | 25,861 | 13,822 | 12,039 | 87 | 491 | 1,665 | 22,333 | 1,237 | 229,479,283 |
| **2004** | 25,247 | 13,483 | 11,764 | 79 | 493 | 1,680 | 21,717 | 1,240 | 232,153,496 |
| **2005** | 25,313 | 13,614 | 11,699 | 89 | 491 | 1,641 | 21,767 | 1,295 | 234,997,553 |
| **2006** | 24,997 | 13,422 | 11,575 | 77 | 491 | 1,693 | 21,399 | 1,312 | 237,863,203 |
| **2007** | 24,984 | 13,576 | 11,408 | 83 | 553 | 1,636 | 21,291 | 1,397 | 240,549,592 |
| **2008** | 24,752 | 13,538 | 11,214 | 80 | 546 | 1,587 | 21,102 | 1,406 | 243,186,582 |
| **2009** | 24,913 | 13,754 | 11,159 | 95 | 576 | 1,737 | 20,953 | 1,528 | 245,683,948 |
| **2010** | 24,842 | 13,604 | 11,238 | 86 | 590 | 1,771 | 20,807 | 1,546 | 247,518,325 |
| **2011** | 24,806 | 13,928 | 10,878 | 80 | 614 | 1,765 | 20,749 | 1,563 | 250,390,811 |
| **2012** | 24,853 | 13,835 | 11,018 | 88 | 647 | 1,703 | 20,702 | 1,657 | 252,769,942 |
| **2013** | 24,603 | 13,755 | 10,848 | 96 | 661 | 1,711 | 20,374 | 1,716 | 255,039,716 |
| **2014** | 24,893 | 13,899 | 10,994 | 84 | 698 | 1,848 | 20,449 | 1,742 | 257,789,101 |
| **2015** | 24,707 | 14,047 | 10,660 | 117 | 757 | 1,814 | 20,244 | 1,703 | 260,402,033 |
| **2016** | 25,016 | 14,096 | 10,920 | 109 | 768 | 1,800 | 20,467 | 1,810 | 262,152,444 |
| **2017** | 25,254 | 14,433 | 10,821 | 107 | 745 | 1,913 | 20,560 | 1,875 | 264,697,626 |
| **2018** | 25,410 | 14,751 | 10,659 | 117 | 821 | 1,931 | 20,530 | 1,961 | 266,281,990 |
| **2019** | 25,463 | 14,563 | 10,900 | 119 | 843 | 1,890 | 20,585 | 1,992 | 267,668,677 |
| **2020** | 26,943 | 15,667 | 11,276 | 138 | 926 | 1,969 | 21,604 | 2,256 | 269,190,697 |
| **Total** | 560,469 | 308,815 | 251,654 | 2,050 | 13,435 | 38,881 | 470,986 | 34,046 | 540,964,9211 |

**100% 55.09% 44.91% 0.37% 2.40% 6.94% 84.01% 6.07%**

**Supplementary Table 2.** NHL-related mortality stratified by place of death in the United States from 1999 to 2020.

| **Year** | **Medical Facility** | **Nursing Home/Long-term Care Facility** | **Hospices** | **Home** | **Other** |
| --- | --- | --- | --- | --- | --- |
| **1999** | 15,142 | 3,939 | Missing | 7,474 | 1,124 |
| **2000** | 14,458 | 4,025 | Missing | 7,469 | 1,131 |
| **2001** | 14,163 | 4,043 | Missing | 7,078 | 1,219 |
| **2002** | 13,723 | 4,051 | Missing | 7,272 | 1,281 |
| **2003** | 13,225 | 3,959 | 104 | 7,144 | 1,351 |
| **2004** | 12,630 | 3,957 | 198 | 6,949 | 1,442 |
| **2005** | 12,341 | 3,967 | 586 | 7,109 | 1,237 |
| **2006** | 11,974 | 3,772 | 838 | 7,163 | 1,188 |
| **2007** | 11,809 | 4,015 | 1,025 | 6,925 | 1,177 |
| **2008** | 11,324 | 3,728 | 1,275 | 6,971 | 1,080 |
| **2009** | 10,875 | 3,898 | 1,370 | 7,047 | 1,151 |
| **2010** | 10,644 | 3,721 | 1,720 | 7,483 | 1,255 |
| **2011** | 10,487 | 3,725 | 1,950 | 7,436 | 1,194 |
| **2012** | 10,080 | 3,504 | 2,256 | 7,769 | 1,229 |
| **2013** | 9,727 | 3,441 | 2,317 | 7,863 | 1,240 |
| **2014** | 9,954 | 3,462 | 2,621 | 7,869 | 968 |
| **2015** | 9,554 | 3,432 | 2,895 | 8,018 | 796 |
| **2016** | 9,577 | 3,345 | 3,008 | 8,281 | 804 |
| **2017** | 9,583 | 3,420 | 3,105 | 8,271 | 871 |
| **2018** | 9,527 | 3,276 | 3,065 | 8,614 | 924 |
| **2019** | 9,638 | 3,271 | 3,169 | 8,453 | 931 |
| **2020** | 9,450 | 2,813 | 2,621 | 10,983 | 1,073 |
| **Total** | 249,885 | 80,764 | 34,123 | 169,641 | 24,666 |

**Supplementary Table 3.** Summary APCs of NHL-related AAMR per 100,000 in the United States from 1999 to 2020.

| **Interval of Years** | **APC (95% CI)** |
| --- | --- |
| **Overall** |  |
| 1999-2004 | -3.22(-4.11 to -2.79) |
| 2004-2018 | -2.15 (-2.28 to -2.02) |
| 2018-2020 | 0.89 (-0.55 to 1.64) |
| **Male** |  |
| 1999-2004 | -3.00 (-4.56 to -2.30) |
| 2004-2016 | -2.05 (-2.39 to -1.48) |
| 2016-2020 | 11.06 (-1.27 to 1.26) |
| **Female** |  |
| 1999-2004 | -3.39 (-4.72 to -2.76) |
| 2004-2018 | -2.57 (-2.87 to -2.18) |
| 2018-2020 | 0.61 (-1.68 to 1.64) |
| **NH American Indian or Alaska Native** |  |
| 1999-2020 | -1.44 (-2.32 to -0.46) |
| **NH Asian or Pacific Islander** |  |
| 1999-2020 | -1.72 (-1.96 to -1.44) |
| **NH Black or African American** |  |
| 1999-2005 | -3.77 (-7.81 to -2.30) |
| 2005-2020 | -1.40 (-1.77 to -0.21) |
| **NH White** |  |
| 1999-2004 | -3.06 (-3.98 to -2.64) |
| 2004-2018 | -2.12 (-2.24 to -1.97) |
| 2018-2020 | 1.33 (-0.17 to 2.16) |
| **Hispanic or Latino** |  |
| 1999-2018 | -1.77 (-3.24 to -1.03) |
| 2018-2020 | 3.11 (-1.61 to 5.79) |

**Supplementary Table 4.** Overall and sex-stratified NHL-related AAMR per 100,000 in the United States from 1999 to 2020.

| **Age-Adjusted Rate (95% CI)** | | | |
| --- | --- | --- | --- |
| **Year** | **Men** | **Women** | **Overall** |
| **1999** | 16.45 (16.18 - 16.72) | 10.27 (10.09 - 10.45) | 12.86 (12.71 - 13.01) |
| **2000** | 15.78 (15.51 - 16.04) | 10.09 (9.92 - 10.27) | 12.46 (12.31 - 12.61) |
| **2001** | 15.31 (15.05 - 15.57) | 9.61 (9.44 - 9.78) | 11.98 (11.84 - 12.13) |
| **2002** | 14.98 (14.72 - 15.23) | 9.4 (9.23 - 9.56) | 11.72 (11.58 - 11.86) |
| **2003** | 14.58 (14.33 - 14.83) | 9.02 (8.86 - 9.18) | 11.34 (11.20 - 11.48) |
| **2004** | 13.94 (13.70 - 14.18) | 8.7 (8.54 - 8.85) | 10.91 (10.77 - 11.04) |
| **2005** | 13.84 (13.60 - 14.07) | 8.5 (8.34 - 8.65) | 10.73 (10.60 - 10.87) |
| **2006** | 13.33 (13.10 - 13.56) | 8.3 (8.15 - 8.46) | 10.43 (10.30 - 10.56) |
| **2007** | 13.18 (12.95 - 13.40) | 8.02 (7.87 - 8.17) | 10.24 (10.11 - 10.37) |
| **2008** | 12.89 (12.67 - 13.11) | 7.78 (7.63 - 7.92) | 9.93 (9.81 - 10.05) |
| **2009** | 12.8 (12.58 - 13.01) | 7.61 (7.47 - 7.75) | 9.82 (9.70 - 9.94) |
| **2010** | 12.42 (12.21 - 12.63) | 7.53 (7.38 - 7.67) | 9.63 (9.51 - 9.75) |
| **2011** | 12.37 (12.16 - 12.57) | 7.17 (7.04 - 7.31) | 9.4 (9.28 - 9.52) |
| **2012** | 11.94 (11.74 - 12.14) | 7.12 (6.99 - 7.26) | 9.17 (9.05 - 9.28) |
| **2013** | 11.57 (11.38 - 11.77) | 6.85 (6.72 - 6.99) | 8.89 (8.78 - 9.00) |
| **2014** | 11.38 (11.18 - 11.57) | 6.77 (6.65 - 6.90) | 8.77 (8.66 - 8.88) |
| **2015** | 11.2 (11.01 - 11.38) | 6.43 (6.31 - 6.56) | 8.51 (8.40 - 8.62) |
| **2016** | 10.95 (10.76 - 11.13) | 6.43 (6.31 - 6.55) | 8.39 (8.28 - 8.49) |
| **2017** | 10.98 (10.80 - 11.17) | 6.28 (6.15 - 6.40) | 8.31 (8.20 - 8.41) |
| **2018** | 10.91 (10.73 - 11.09) | 6.03 (5.91 - 6.14) | 8.17 (8.07 - 8.27) |
| **2019** | 10.5 (10.33 - 10.67) | 6.04 (5.93 - 6.16) | 8 (7.90 - 8.10) |
| **2020** | 11.06 (10.88 - 11.23) | 6.14 (6.03 - 6.26) | 8.26 (8.16 - 8.36) |
| **Total** | 12.62 (12.57 - 12.66) | 7.58 (7.55 - 7.61) | 9.74 (9.71 - 9.77) |

**Supplementary Table 5.** NHL-related AAMR per 100,000 stratified by Race in the United States from 1999 to 2020.

|  | **Age-Adjusted Rate (95% CI)** | | | |  |
| --- | --- | --- | --- | --- | --- |
| **Year** | **NH American Indian or Alaska Native** | **NH Asian or Pacific Islander** | **NH Black or African American** | **NH White** | **Hispanic or Latino** |
| **1999** | 7.26 (5.58 - 9.29) | 7.76 (6.97 - 8.56) | 9.68 (9.24 - 10.12) | 13.42 (13.25 - 13.59) | 9.61 (9.03 - 10.20) |
| **2000** | 9.22 (7.32 - 11.46) | 7.84 (7.06 - 8.61) | 8.37 (7.96 - 8.78) | 13.11 (12.95 - 13.28) | 9.38 (8.81 - 9.95) |
| **2001** | 9.61 (7.61 - 11.61) | 7.33 (6.61 - 8.05) | 8.66 (8.24 - 9.07) | 12.62 (12.46 - 12.79) | 8.75 (8.21 - 9.29) |
| **2002** | 4.82 (3.54 - 6.41) | 6.60 (5.95 - 7.26) | 8.29 (7.89 - 8.69) | 12.37 (12.21 - 12.53) | 8.84 (8.31 - 9.37) |
| **2003** | 7.58 (5.98 - 9.48) | 7.31 (6.63 - 7.98) | 7.81 (7.43 - 8.20) | 11.96 (11.80 - 12.12) | 8.38 (7.88 - 8.88) |
| **2004** | 7.22 (5.64 - 9.10) | 6.99 (6.35 - 7.64) | 7.84 (7.46 - 8.23) | 11.49 (11.34 - 11.65) | 7.96 (7.49 - 8.44) |
| **2005** | 7.03 (5.55 - 8.79) | 6.41 (5.82 - 7.00) | 7.36 (6.99 - 7.72) | 11.38 (11.23 - 11.54) | 8.28 (7.81 - 8.76) |
| **2006** | 6.98 (5.42 - 8.85) | 6.18 (5.61 - 6.75) | 7.44 (7.08 - 7.81) | 11.05 (10.90 - 11.20) | 7.80 (7.36 - 8.25) |
| **2007** | 7.06 (5.53 - 8.88) | 6.61 (6.04 - 7.18) | 7.01 (6.66 - 7.36) | 10.81 (10.67 - 10.96) | 8.01 (7.57 - 8.46) |
| **2008** | 6.24 (4.86 - 7.88) | 6.31 (5.76 - 6.86) | 6.65 (6.31 - 6.98) | 10.58 (10.43 - 10.72) | 7.62 (7.20 - 8.05) |
| **2009** | 7.02 (5.59 - 8.70) | 6.39 (5.85 - 6.93) | 7.15 (6.80 - 7.49) | 10.34 (10.19 - 10.48) | 7.95 (7.53 - 8.37) |
| **2010** | 6.62 (5.22 - 8.29) | 6.14 (5.63 - 6.66) | 7.19 (6.85 - 7.54) | 10.14 (10.00 - 10.28) | 7.83 (7.41 - 8.24) |
| **2011** | 5.62 (4.40 - 7.08) | 6.10 (5.61 - 6.60) | 6.97 (6.63 - 7.30) | 9.92 (9.78 - 10.05) | 7.49 (7.10 - 7.88) |
| **2012** | 6.27 (4.96 - 7.81) | 5.94 (5.47 - 6.41) | 6.53 (6.21 - 6.85) | 9.70 (9.57 - 9.84) | 7.48 (7.10 - 7.86) |
| **2013** | 6.30 (5.04 - 7.78) | 5.78 (5.33 - 6.23) | 6.35 (6.04 - 6.66) | 9.40 (9.27 - 9.53) | 7.39 (7.03 - 7.76) |
| **2014** | 5.21 (4.10 - 6.52) | 5.65 (5.22 - 6.08) | 6.62 (6.31 - 6.93) | 9.29 (9.16 - 9.42) | 7.19 (6.84 - 7.54) |
| **2015** | 7.04 (5.70 - 8.37) | 5.77 (5.35 - 6.18) | 6.36 (6.06 - 6.66) | 9.02 (8.89 - 9.15) | 6.55 (6.22 - 6.87) |
| **2016** | 6.21 (5.00 - 7.43) | 5.59 (5.19 - 6.00) | 6.03 (5.74 - 6.32) | 8.95 (8.83 - 9.08) | 6.88 (6.55 - 7.21) |
| **2017** | 5.45 (4.38 - 6.53) | 5.10 (4.73 - 5.47) | 6.35 (6.06 - 6.64) | 8.87 (8.74 - 8.99) | 6.68 (6.36 - 6.99) |
| **2018** | 6.02 (4.89 - 7.15) | 5.39 (5.02 - 5.76) | 6.21 (5.92 - 6.49) | 8.65 (8.53 - 8.77) | 6.71 (6.40 - 7.02) |
| **2019** | 5.71 (4.65 - 6.77) | 5.26 (4.90 - 5.62) | 5.97 (5.69 - 6.25) | 8.54 (8.42 - 8.66) | 6.49 (6.19 - 6.78) |
| **2020** | 6.66 (5.51 - 7.80) | 5.53 (5.17 - 5.89) | 6.03 (5.76 - 6.31) | 8.84 (8.72 - 8.96) | 7.13 (6.82 - 7.43) |
| **Total** | 6.55 (6.25 - 6.84) | 6.04 (5.94 - 6.14) | 7.03 (6.95 - 7.10) | 10.34 (10.32 - 10.37) | 7.53 (7.44 - 7.61) |

**Supplementary Table 6.** NHL-related AAMR per 100,000 stratified by Urban-Rural classification in the United States from 1999 to 2020.

|  | **Age-Adjusted Rate (95% CI)** |  |
| --- | --- | --- |
| **Year** | **Metropolitan** | **Nonmetropolitan** |
| 1999 | 12.93 (12.76–13.09) | 12.59 (12.24–12.93) |
| 2000 | 12.39 (12.22–12.55) | 12.73 (12.38–13.07) |
| 2001 | 11.91 (11.75–12.07) | 12.33 (11.99–12.67) |
| 2002 | 11.7 (11.55–11.86) | 11.89 (11.55–12.22) |
| 2003 | 11.25 (11.1–11.4) | 11.75 (11.42–12.08) |
| 2004 | 10.85 (10.7–11.0) | 11.13 (10.81–11.45) |
| 2005 | 10.65 (10.5–10.8) | 11.16 (10.84–11.48) |
| 2006 | 10.38 (10.23–10.52) | 10.69 (10.38–10.99) |
| 2007 | 10.12 (9.98–10.26) | 10.73 (10.42–11.04) |
| 2008 | 9.83 (9.69–9.96) | 10.43 (10.12–10.73) |
| 2009 | 9.72 (9.59–9.86) | 10.2 (9.9–10.49) |
| 2010 | 9.53 (9.4–9.67) | 10.1 (9.81–10.4) |
| 2011 | 9.24 (9.11–9.37) | 10.12 (9.82–10.41) |
| 2012 | 9.01 (8.89–9.14) | 9.9 (9.61–10.19) |
| 2013 | 8.78 (8.65–8.9) | 9.37 (9.09–9.65) |
| 2014 | 8.68 (8.56–8.8) | 9.21 (8.93–9.48) |
| 2015 | 8.38 (8.26–8.5) | 9.1 (8.83–9.37) |
| 2016 | 8.27 (8.16–8.39) | 9.05 (8.78–9.32) |
| 2017 | 8.19 (8.07–8.3) | 8.85 (8.59–9.11) |
| 2018 | 8.06 (7.95–8.17) | 8.71 (8.46–8.97) |
| 2019 | 7.88 (7.77–7.99) | 8.66 (8.41–8.92) |
| 2020 | 8.1 (7.99–8.21) | 9.25 (8.99–9.51) |

**Supplementary Table 7.** NHL-related AAMR per 100,000 stratified by state in the United States from 1999 to 2020.

| **State** | **Age-Adjusted Rate (95% CI)** |
| --- | --- |
| **Alabama** | 8.98 (8.79 - 9.18) |
| **Alaska** | 9.12 (8.43 - 9.82) |
| **Arizona** | 8.55 (8.38 - 8.71) |
| **Arkansas** | 9.6 (9.35 - 9.86) |
| **California** | 9.57 (9.49 - 9.64) |
| **Colorado** | 8.94 (8.73 - 9.15) |
| **Connecticut** | 9.64 (9.42 - 9.87) |
| **Delaware** | 9.51 (9.05 - 9.96) |
| **District of Columbia** | 8.28 (7.72 - 8.84) |
| **Florida** | 8.88 (8.79 - 8.97) |
| **Georgia** | 8.4 (8.25 - 8.55) |
| **Hawaii** | 8.29 (7.94 - 8.63) |
| **Idaho** | 10.05 (9.68 - 10.43) |
| **Illinois** | 9.86 (9.73 - 9.98) |
| **Indiana** | 10.89 (10.70 - 11.08) |
| **Iowa** | 11.06 (10.81 - 11.32) |
| **Kansas** | 10.33 (10.06 - 10.60) |
| **Kentucky** | 10.65 (10.42 - 10.87) |
| **Louisiana** | 9.54 (9.32 - 9.75) |
| **Maine** | 10.32 (9.95 - 10.68) |
| **Maryland** | 9.24 (9.05 - 9.43) |
| **Massachusetts** | 9.47 (9.30 - 9.64) |
| **Michigan** | 10.87 (10.72 - 11.02) |
| **Minnesota** | 11.22 (11.01 - 11.42) |
| **Mississippi** | 8.85 (8.60 - 9.11) |
| **Missouri** | 10.06 (9.88 - 10.25) |
| **Montana** | 9.68 (9.25 - 10.11) |
| **Nebraska** | 11.09 (10.74 - 11.44) |
| **Nevada** | 8.17 (7.90 - 8.44) |
| **New Hampshire** | 9.71 (9.33 - 10.10) |
| **New Jersey** | 9.67 (9.53 - 9.82) |
| **New Mexico** | 8.24 (7.94 - 8.53) |
| **New York** | 9.41 (9.31 - 9.51) |
| **North Carolina** | 9.18 (9.04 - 9.32) |
| **North Dakota** | 9.96 (9.44 - 10.48) |
| **Ohio** | 11.05 (10.91 - 11.19) |
| **Oklahoma** | 10.86 (10.62 - 11.11) |
| **Oregon** | 10.98 (10.74 - 11.21) |
| **Pennsylvania** | 10.53 (10.41 - 10.65) |
| **Rhode Island** | 10.38 (9.95 - 10.80) |
| **South Carolina** | 8.91 (8.71 - 9.11) |
| **South Dakota** | 10.7 (10.20 - 11.19) |
| **Tennessee** | 10.42 (10.23 - 10.61) |
| **Texas** | 9.32 (9.23 - 9.42) |
| **Utah** | 9.17 (8.85 - 9.48) |
| **Vermont** | 11 (10.43 - 11.58) |
| **Virginia** | 9.05 (8.89 - 9.21) |
| **Washington** | 10.62 (10.43 - 10.80) |
| **West Virginia** | 10.98 (10.65 - 11.30) |
| **Wisconsin** | 10.57 (10.38 - 10.76) |
| **Wyoming** | 9.27 (8.67 - 9.88) |

**Supplementary Table 8.** NHL-related AAMR per 100,000 stratified by census region in the United States from 1999 to 2020.

| **Census Region** | Year | Age-Adjusted Rate (95% CI) |
| --- | --- | --- |
| Census Region 1: Northeast | 1999 | 13.2 (12.87 - 13.54) |
| Census Region 1: Northeast | 2000 | 12.73 (12.40 - 13.06) |
| Census Region 1: Northeast | 2001 | 12.1 (11.78 - 12.42) |
| Census Region 1: Northeast | 2002 | 11.89 (11.58 - 12.21) |
| Census Region 1: Northeast | 2003 | 11.44 (11.14 - 11.75) |
| Census Region 1: Northeast | 2004 | 11.03 (10.73 - 11.33) |
| Census Region 1: Northeast | 2005 | 10.57 (10.28 - 10.86) |
| Census Region 1: Northeast | 2006 | 10.41 (10.12 - 10.70) |
| Census Region 1: Northeast | 2007 | 10.21 (9.92 - 10.49) |
| Census Region 1: Northeast | 2008 | 9.91 (9.63 - 10.19) |
| Census Region 1: Northeast | 2009 | 9.54 (9.27 - 9.81) |
| Census Region 1: Northeast | 2010 | 9.63 (9.35 - 9.90) |
| Census Region 1: Northeast | 2011 | 9.61 (9.34 - 9.88) |
| Census Region 1: Northeast | 2012 | 9.13 (8.87 - 9.39) |
| Census Region 1: Northeast | 2013 | 8.71 (8.46 - 8.97) |
| Census Region 1: Northeast | 2014 | 8.76 (8.50 - 9.01) |
| Census Region 1: Northeast | 2015 | 8.67 (8.42 - 8.92) |
| Census Region 1: Northeast | 2016 | 8.61 (8.36 - 8.86) |
| Census Region 1: Northeast | 2017 | 8.5 (8.26 - 8.75) |
| Census Region 1: Northeast | 2018 | 7.97 (7.74 - 8.21) |
| Census Region 1: Northeast | 2019 | 7.67 (7.44 - 7.90) |
| Census Region 1: Northeast | 2020 | 8.22 (7.99 - 8.45) |
| **TOTAL** |  | 9.82 (9.76 - 9.88) |
| Census Region 2: Midwest | 1999 | 13.86 (13.54 - 14.19) |
| Census Region 2: Midwest | 2000 | 13.72 (13.40 - 14.04) |
| Census Region 2: Midwest | 2001 | 13.12 (12.80 - 13.43) |
| Census Region 2: Midwest | 2002 | 12.62 (12.31 - 12.92) |
| Census Region 2: Midwest | 2003 | 12.1 (11.81 - 12.40) |
| Census Region 2: Midwest | 2004 | 11.71 (11.42 - 12.00) |
| Census Region 2: Midwest | 2005 | 11.59 (11.31 - 11.88) |
| Census Region 2: Midwest | 2006 | 11.12 (10.84 - 11.40) |
| Census Region 2: Midwest | 2007 | 10.96 (10.68 - 11.23) |
| Census Region 2: Midwest | 2008 | 10.79 (10.52 - 11.06) |
| Census Region 2: Midwest | 2009 | 10.8 (10.53 - 11.07) |
| Census Region 2: Midwest | 2010 | 10.51 (10.25 - 10.78) |
| Census Region 2: Midwest | 2011 | 10.5 (10.24 - 10.77) |
| Census Region 2: Midwest | 2012 | 9.9 (9.64 - 10.15) |
| Census Region 2: Midwest | 2013 | 9.56 (9.31 - 9.81) |
| Census Region 2: Midwest | 2014 | 9.61 (9.36 - 9.86) |
| Census Region 2: Midwest | 2015 | 9.41 (9.17 - 9.65) |
| Census Region 2: Midwest | 2016 | 8.99 (8.75 - 9.22) |
| Census Region 2: Midwest | 2017 | 8.94 (8.71 - 9.17) |
| Census Region 2: Midwest | 2018 | 8.99 (8.76 - 9.22) |
| Census Region 2: Midwest | 2019 | 8.98 (8.75 - 9.21) |
| Census Region 2: Midwest | 2020 | 9.22 (8.99 - 9.45) |
| **TOTAL** |  | 10.64 (10.59 - 10.70) |
| Census Region 3: South | 1999 | 12.19 (11.94 - 12.44) |
| Census Region 3: South | 2000 | 11.72 (11.48 - 11.96) |
| Census Region 3: South | 2001 | 11.34 (11.11 - 11.58) |
| Census Region 3: South | 2002 | 11.13 (10.89 - 11.36) |
| Census Region 3: South | 2003 | 10.77 (10.54 - 10.99) |
| Census Region 3: South | 2004 | 10.45 (10.23 - 10.67) |
| Census Region 3: South | 2005 | 10.33 (10.11 - 10.55) |
| Census Region 3: South | 2006 | 10.03 (9.82 - 10.24) |
| Census Region 3: South | 2007 | 9.73 (9.52 - 9.93) |
| Census Region 3: South | 2008 | 9.48 (9.28 - 9.68) |
| Census Region 3: South | 2009 | 9.5 (9.30 - 9.70) |
| Census Region 3: South | 2010 | 9.23 (9.04 - 9.43) |
| Census Region 3: South | 2011 | 8.79 (8.60 - 8.98) |
| Census Region 3: South | 2012 | 8.88 (8.70 - 9.07) |
| Census Region 3: South | 2013 | 8.69 (8.51 - 8.87) |
| Census Region 3: South | 2014 | 8.37 (8.20 - 8.55) |
| Census Region 3: South | 2015 | 8.06 (7.89 - 8.23) |
| Census Region 3: South | 2016 | 8.1 (7.93 - 8.27) |
| Census Region 3: South | 2017 | 7.95 (7.79 - 8.12) |
| Census Region 3: South | 2018 | 7.89 (7.73 - 8.05) |
| Census Region 3: South | 2019 | 7.78 (7.62 - 7.94) |
| Census Region 3: South | 2020 | 7.91 (7.75 - 8.07) |
| **TOTAL** |  | 9.27 (9.23 - 9.31) |
| Census Region 4: West | 1999 | 12.54 (12.21 - 12.87) |
| Census Region 4: West | 2000 | 12.05 (11.73 - 12.38) |
| Census Region 4: West | 2001 | 11.75 (11.44 - 12.07) |
| Census Region 4: West | 2002 | 11.59 (11.28 - 11.90) |
| Census Region 4: West | 2003 | 11.3 (10.99 - 11.60) |
| Census Region 4: West | 2004 | 10.66 (10.36 - 10.95) |
| Census Region 4: West | 2005 | 10.57 (10.28 - 10.86) |
| Census Region 4: West | 2006 | 10.26 (9.98 - 10.54) |
| Census Region 4: West | 2007 | 10.32 (10.04 - 10.60) |
| Census Region 4: West | 2008 | 9.8 (9.53 - 10.07) |
| Census Region 4: West | 2009 | 9.51 (9.25 - 9.77) |
| Census Region 4: West | 2010 | 9.38 (9.12 - 9.63) |
| Census Region 4: West | 2011 | 9.11 (8.86 - 9.36) |
| Census Region 4: West | 2012 | 8.96 (8.71 - 9.20) |
| Census Region 4: West | 2013 | 8.69 (8.45 - 8.93) |
| Census Region 4: West | 2014 | 8.58 (8.35 - 8.81) |
| Census Region 4: West | 2015 | 8.27 (8.04 - 8.49) |
| Census Region 4: West | 2016 | 8.16 (7.94 - 8.39) |
| Census Region 4: West | 2017 | 8.21 (7.99 - 8.43) |
| Census Region 4: West | 2018 | 7.98 (7.76 - 8.19) |
| Census Region 4: West | 2019 | 7.76 (7.55 - 7.97) |
| Census Region 4: West | 2020 | 8.07 (7.86 - 8.28) |
| **TOTAL** |  | 9.49 (9.44 - 9.54) |

**Supplementary Table 9.** NHL-related deaths per 100,000 stratified by top 15 underlying causes of death in the United States from 1999 to 2020

| **UCD - 15 Leading Causes of Death** | Deaths (1999-2020) |
| --- | --- |
| **Malignant neoplasms (C00-C97)** | 488,976 |
| **Diseases of heart (I00-I09,I11,I13,I20-I51)** | 26,384 |
| **Chronic lower respiratory diseases (J40-J47)** | 5,137 |
| **Cerebrovascular diseases (I60-I69)** | 3,463 |
| **Human immunodeficiency virus (HIV) disease (B20-B24)** | 3,012 |
| **Diabetes mellitus (E10-E14)** | 2,419 |
| **Accidents (unintentional injuries) (V01-X59,Y85-Y86)** | 2,284 |
| **In situ neoplasms, benign neoplasms and neoplasms of uncertain or unknown behavior (D00-D48)** | 1,828 |
| **Alzheimer disease (G30)** | 1,602 |
| **Nephritis, nephrotic syndrome and nephrosis (N00-N07,N17-N19,N25-N27)** | 1,556 |
| **Chronic liver disease and cirrhosis (K70,K73-K74)** | 1,203 |
| **Essential hypertension and hypertensive renal disease (I10,I12,I15)** | 1,124 |
| **Pneumonitis due to solids and liquids (J69)** | 1,110 |
| **COVID-19 (U07.1)** | 1,061 |
| **Influenza and pneumonia (J09-J18)** | 834 |
